# Daily activity budgets reveal a quasi-flightless stage during non-breeding in Hawaiian albatrosses

**DOI:** 10.1186/s40462-014-0023-4

**Published:** 2014-11-19

**Authors:** Sarah E Gutowsky, Lee FG Gutowsky, Ian D Jonsen, Marty L Leonard, Maura B Naughton, Marc D Romano, Scott A Shaffer

**Affiliations:** Biology Department, Dalhousie University, Halifax, NS Canada; Fish Ecology & Conservation Physiology Lab, Carleton University, Ottawa, ON Canada; Department of Biological Sciences, Macquarie University, Sydney, NSW Australia; USFWS, Pacific Region, Migratory Birds and Habitat Programs, Portland, OR USA; Department of Biological Sciences, San Jose State University, San Jose, CA USA; Institute of Marine Sciences, University of California, Santa Cruz, CA USA

**Keywords:** Activity budget, Behaviour, Biologging, Flight, Migration, Moult, Seabirds, North Pacific, Non-breeding, Overwinter

## Abstract

**Background:**

Animals adjust activity budgets as competing demands for limited time and energy shift across life history phases. For far-ranging migrants and especially pelagic seabirds, activity during breeding and migration are generally well studied but the “overwinter” phase of non-breeding has received less attention. Yet this is a critical time for recovery from breeding, plumage replacement and gaining energy stores for return migration and the next breeding attempt. We aimed to identify patterns in daily activity budgets (i.e. time in flight, floating on the water’s surface and active foraging) and associated spatial distributions during overwinter for the laysan *Phoebastria immutabilis* and black-footed *P. nigripes* albatrosses using state-space models and generalized additive mixed-effects models (GAMMs). We applied these models to time-series of positional and immersion-state data from small light- and conductivity-based data loggers.

**Results:**

During overwinter, both species exhibited a consistent ‘quasi-flightless’ stage beginning *c.* 30 days after initiating migration and lasting *c.* 40 days, characterized by frequent long bouts of floating, very little sustained flight, and infrequent active foraging. Minimal daily movements were made within localized areas during this time; individual laysan albatross concentrated into the northwest corner of the Pacific while black-footed albatross spread widely across the North Pacific Ocean basin. Activity gradually shifted toward increased time in flight and active foraging, less time floating, and greater daily travel distances until colony return *c.* 155 days after initial departure.

**Conclusions:**

Our results demonstrate that these species make parallel adjustments to activity budgets at a daily time-scale within the overwinter phase of non-breeding despite different at-sea distributions and phenologies. The ‘quasi-flightless’ stage likely reflects compromised flight from active wing moult while the subsequent increase in activity may occur as priorities shift toward mass gain for breeding. The novel application of a GAMM-based approach used in this study offers the possibility of identifying population-level patterns in shifting activity budgets over extended periods while allowing for individual-level variation in the timing of events. The information gained can also help to elucidate the whereabouts of areas important at different times across life history phases for far-ranging migrants.

## Background

As resource needs and availability change across life history phases, animals must adjust activity budgets to spend proportionally more or less time engaged in different activities with varying potential for net energy gain. Far-ranging migrants in particular make drastic adjustments to daily activity budgets as they move between vastly separated areas important at different phases in the annual cycle [1,2]. Marine species can present a challenge in that comprehensively understanding activity budgets requires knowledge of behaviour and distributions for regions that may be separated by thousands of kilometres, often in inaccessible pelagic locations. For species that rely on land to breed, the breeding period is typically well studied while much less is known of activities during the non-breeding period, despite the important influence of this time on population dynamics [3]. Seabirds, for example, need the non-breeding period to recover from the demands of raising offspring, replace plumage and prepare for the next migratory journey and breeding attempt [3-5].

Among the world’s most impressive migrants, larger members of the ‘tube-nosed seabirds’ (shearwaters and albatrosses of the O. *Procellariiformes*) are relatively well studied throughout breeding, as well as certain aspects of non-breeding. Some of the swiftest and most far-reaching migrations known are accomplished by tubenoses (but see [6]). For example, sooty shearwaters *Puffinus griseus* spend extended periods engaged in flight and little time resting while in migratory transit across transequatorial routes around the Pacific [7]. These birds travel at rates of up to 1000 km/day, accomplishing round-trip journeys of over 70,000 km between breeding and non-breeding overwintering grounds [7]. However, as for most seabirds, daily activity and potential energetic needs or constraints within the “overwinter” phase of non-breeding have been examined in comparatively little detail relative to these often spectacular outbound and inbound migrations.

Constant advancements in biologging technology and data analysis are allowing increasingly detailed investigations into the at-sea activity of seabirds during different phases of breeding and non-breeding (e.g. [8-19]). Many shearwater and albatross species are ideal for deployment and retrieval of biologging devices because of their ties to a predictable ‘central place’ [20] at convenient densities for study when nesting. External temperature or wet/dry immersion loggers allow estimation of the allocation of time toward different activities. For example, prolonged warm/dry periods can indicate bouts of sustained flight, prolonged cold/wet periods indicate time on the water’s surface (e.g., [8,10]), and brief and continuous wet/dry transitions indicate ‘active foraging’ (e.g., [17]). In combination with internal stomach temperature logger data, it is possible to estimate the relative potential net energy gained when engaged in each activity. For example, while active foraging bouts have been found to account for the majority of prey ingestion, some prey can still be captured when floating (a ‘sit-and-wait’ foraging strategy) and also occasionally during sustained flight bouts (a ‘fly-and-forage’ strategy [9,11]).

Using these techniques, the overwinter phase has been broadly characterized for some species by reduced flight activity and frequent long bouts on the water relative to all other life history phases (e.g. [13,14,19]). This may be due to a combination of lower energetic demands from the lack of a central place constraint to the nest and locally productive foraging conditions (e.g., four albatross *spp*. [14]), along with possible constraints to mobility from moulting (e.g., sooty shearwater [18]). However, the non-breeding period can be lengthy (e.g. *c.* 200 days for sooty shearwaters [7] or *c.* 18-months for grey-headed albatross *Thalassarche chrysostoma* [21]). For many species, the vast majority of this time is spent in overwintering areas between swift migratory phases. Because energetic priorities and constraints inevitably shift within this long timespan, we could expect that average overwinter activity budgets likely mask major short-term changes in activity during this important time. Generalizations may conceal fine-scale modifications to activity, and may make identification of more sensitive time periods or important at-sea areas challenging.

The present study aimed to objectively identify patterns in activity and associated at-sea distributions across the overwinter phase of non-breeding using two North Pacific tubenoses as model species. The laysan (*Phoebastria immutabilis*, LAAL) and black-footed (*P. nigripes*, BFAL) albatross range widely across the North Pacific during non-breeding after they have vacated breeding colonies found mostly in the Northwestern Hawaiian Islands [22]. These two species differ in diet and habitat preferences but breed sympatrically and are similar in size and breeding phenology [22,23]. A number of anthropogenic threats have lead to LAAL and BFAL listing as ‘Near Threatened’ [24]. Much is known from biologging studies of habitat use and behaviour of both species during breeding [25-29] and of at-sea distributions during non-breeding [29-34]. For the largest colony of both species at Midway Atoll National Wildlife Refuge, (herein ‘Midway’; 70% of worldwide LAAL and 35% of BFAL [22]), however, non-breeding activity and habitat use are mostly undocumented beyond coarse-scale distributions of BFAL during the month of August [34].

Using small light- and conductivity-based archival data loggers, we examined daily activity budgets across the entire non-breeding season of LAAL and BFAL from Midway. Specifically, we identified patterns in time allocation between sustained flight, floating and active foraging, and associated distributions, by applying state-space models [35,36] and generalized additive mixed-effects models [37] to time-series of positional and immersion-state data. This allowed evaluation of patterns in activity not only by broad phases of non-breeding but also at a daily time-scale, elucidating new insights into population-level patterns and commonalities among species in the likely energetic constraints faced during this time.

## Methods

### Logger deployment

**Figure 1:**
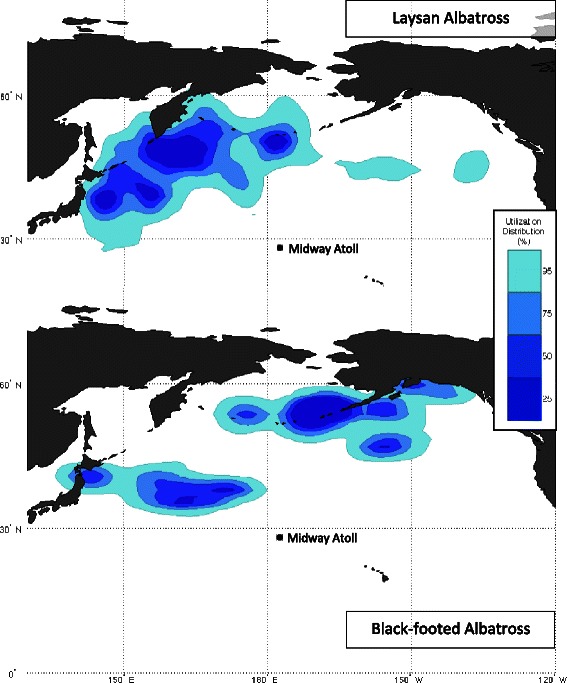
**Overwinter destinations of laysan and black-footed albatross from Midway.** Kernel density analysis of 95%, 75%, 50% and 25% utilization distribution (UD) contours, in increasingly darker shades of blue, for GLS-tracked laysan albatross (*n* = 18, top panel) and black-footed albatross (*n* = 15, bottom panel) during the overwinter phase of non-breeding in 2008, 2009, 2011 and 2012. The solid black circle indicates the colony at Midway Atoll National Wildlife Refuge.

Fieldwork was conducted over five field seasons (2008, 2009, 2010, 2011 and 2012) at Sand Island, Midway Atoll (28.12°N, 177.23°W, Figure 1). Midway is home to roughly 408,000 breeding pairs of LAAL and 22,000 pairs of BFAL [22]. We deployed leg-mounted geolocation-immersion loggers (herein ‘GLS’; Lotek LAT2500, Lotek Wireless Inc, St John's, Newfoundland, CA) on equal numbers of opportunistically selected breeding adults of both species (sex unknown) during incubation or early chick rearing (between December and March) and recovered GLS during incubation in the subsequent breeding season (between early-December and early-January). GLS were mounted on a plastic leg band using UV resistant cable ties and quick-setting epoxy (logger + attachment ~6 g, <1% body mass; well below the recommended limit for albatrosses [38]). While it was not possible to formally assess tag effects in this study, deployments and retrievals took no longer than 10 min and did not appear to interfere with nesting behaviour. Further, visible inspection upon retrieval indicated that attachments did not cause any physical harm. GLS deployments at other colonies of LAAL have resulted in no detectable short-term effects on reproductive success [29]. Of 119 GLS deployed over five seasons, 77% were recovered. Upon download, 62% of those recovered revealed technological failures causing appreciably spurious or missing light or immersion data, yielding 35 GLS with complete concurrent time series of both location and immersion data for this study. All tags recovered from the 2010 deployment season (*n* = 20) failed to produce reliable immersion data and were not used in subsequent analyses.

All recovered and functional GLS recorded light levels at 10-minute intervals to estimate daily locations and saltwater immersion to estimate on/off water activity patterns (determined by conductivity between two external pins). GLS recorded instantaneous immersion state (wet or dry) at a programmed interval and produced time series of states with resolutions between 32 and 100 seconds depending on tag programming in the year of deployment (2008: 100-sec, LAAL *n* = 3, BFAL *n* = 4; 2009: 90-sec, LAAL *n* = 8, BFAL *n* = 6; 2011/2012: 32-sec, LAAL *n* = 9, BFAL *n* = 5). Immersion state changes occurring in <90 seconds were excluded, ensuring all time series reflect similar behavioural changes [17]. Light data was processed with automated template fitting software, producing a single location per day using sunrise and sunset times and estimate latitude from day length and longitude from the time of local noon/midnight [39]. The accuracy of latitude estimates during equinox periods is unavoidably compromised, as day length depends only weakly on latitude at this time [39]. For this study, locations on 15 days of either side of the fall equinox were excluded based on consistently suspect latitude estimates.

### Positional data processing

Cloud cover, feather shading or large daily travel distances can further compromise light signals causing short periods of spurious or missing locations [40]. Unrealistic location estimates are often discarded from a dataset based on subjective criteria [41]. We used recently developed hierarchical time-series state-space models (SSMs) estimated with Bayesian techniques to avoid unnecessary data loss. This approach comprises two probabilistic components: a process model of the biological mechanisms influencing locations and an observation model of how the location estimates were obtained. SSMs correct observed locations for tag error and biological realism to make inferences about the true ‘hidden state’ or locations [35,36]. Estimates of tag error have been derived elsewhere by ‘double-tagging’ experiments in which LAAL and BFAL carried both GLS and higher-accuracy satellite Platform Terminal Transmitter (PTT) tags (LAAL longitude error SD = 1.9° and latitude error SD = 1.2°, BFAL longitude error SD = 3.8° and latitude error SD = 1.9°; [40] further refined in [33]). Latitude estimates in these studies were derived using an algorithm that matches remotely-sensed SST-gradients to SST data recorded on-board the GLS. We did not have reliable SST data for all GLS in this study and therefore observed latitudes are more likely to have errors similar to those estimated for longitude. We took a conservative approach by fixing the SSM tag error parameter estimates for both latitude and longitude of both species equal to the maximum estimated error for longitude in [33]. Positions falling over continental landmasses were constrained toward the marine environment in the SSM by a land mask. The SSM was fitted using Markov Chain Monte Carlo (MCMC) sampling. For each bird, two independent and parallel MCMC chains each of length 100,000 were run and a sample of 2,000 from the joint posterior probability distribution was obtained by discarding the first 80,000 iterations and retaining every 20^th^ of the remaining iterations. MCMC algorithm convergence was assessed using the ratio of variances for parameters between the retained MCMC chains (the potential scale reduction factor or *R-hat* statistic); when models are well converged, the values are near 1. The final SSM-processed once-daily true-position estimates were obtained from the mean of appropriately converged posterior distributions [35].

### Individual seasonal phenology

We estimated the timing of non-breeding departure and return based on known travel rates using patterns in positional data and great circle distance to the colony. Both species are known to travel >30 km/h on foraging trips from the colony with an unlikely but not impossible maximum daily distance travelled of 720 km [28]. The date of initiation of the non-breeding season (i.e. definitively no longer visiting the colony) was determined as the first day an individual was estimated >720 km from the colony with all subsequent locations increasingly distant without return. Similarly, we determined the final day of the non-breeding season as the first day with distance to the colony <720 km with a clear pattern of decreasing distance to the colony before this date and locations indicating potential colony visits after this date.

We delineated the three phases of non-breeding for each bird through visual inspection of daily movement patterns and individual non-breeding phenology: outbound transit (series of consecutive movements following departure directed away from the colony with daily travel rates >100 km/day), overwinter (beginning with the first prolonged series of days with decreased travel rate and directed movements), and inbound transit (series of travel days terminating on the probable colony return date as determined above, Table 1). For two LAAL, initiation of the inbound transit phase overlapped the end of the equinox window; therefore daily activity parameters for these birds are only used to describe the full non-breeding period.

**Table 1:** **Phenology of non-breeding season phases for laysan (** ***n*** = 20, or if *, ***n*** **= 18) and black-footed (** ***n*** **= 15) albatross from Midway Atoll (mean ± SD), 2008-2012**

	**Laysan albatross**	**Black-footed albatross**
Colony departure	29-Jun ±16 days	25-Jun ±14 days
Duration of outbound transit	10 ± 6 days	12 ± 8 days
Overwinter arrival	10-Jul ±16 days	02-Jul ±11 days
Duration of overwinter	125 ± 18 days*	126 ± 21 days
Overwinter departure	12-Nov ±5 days*	05-Nov ±8 days
Duration of inbound transit	9 ± 4 days*	10 ± 8 days
Colony return	18-Nov ±13 days	16-Nov ±14 days

We examined patterns of at-sea distribution among individuals with kernel density analysis [42] applied to SSM-processed locations using software written in Matlab (MathWorks Inc, USA; IKNOS Toolbox). The geographic coordinates of each bird location for each phase were transformed to Cartesian coordinates using a Lambert Cylindrical Equal Area projection and 2D Gaussian kernel densities computed on a 0.25° × 0.25° grid. We estimated the smoothing parameter (h) using an adaptive method to estimate an optimal local value [43]. Each cell was normalized for bird effort by dividing the number of locations within each cell by the number of birds contributing to the cell [28,44]. We divided the density surface into concentric polygons to calculate utilization distribution (UD) contours of 95% (active range), 75%, 50%, and 25% (core hotspot areas).

### Immersion state data processing

We used immersion state time series (wet/dry) to calculate the following parameters related to daily at-sea activity: 1) the number and duration of sustained bouts of flight, floating and active foraging each day (details below) and 2) the proportion of each day spent in sustained flight, floating on the water and actively foraging. Wet or dry intervals that overlapped the cut-off transition between days at midnight were excluded [18,45].

To identify different bout types, we assessed patterns in the immersion state time series. A period of relatively brief and continuous wet-dry transitions resulting from an episode of frequent landing and take-off events from the ocean’s surface can be used as an indicator of ‘active foraging’ in non-diving seabirds (e.g., [9,17]). A small number of these episodes could also indicate other activities including conspecific interactions, but at least reflect periods of active movements and increased energy expenditure [45-47], given birds are alighting from and landing on the water while flying relatively short distances between landings, probably requiring at least some flapping flight. Longer periods of sustained wet or dry states are taken to indicate bouts of prolonged floating on the water’s surface (wet) or flight (dry). The temporal interval breakpoint that separates periods of rapid wet-dry transitions and periods of prolonged wet or dry activity can be identified as a *bout ending criteria* or *BEC* using a maximum-likelihood approach [48]. This approach has been employed widely on diving animals with time-depth recorders (e.g., [49-51]) but much less on non-diving seabirds that do not forage below the first few meters of the ocean surface. Following methods outlined in [17], individual *BEC*s were calculated using the diveMove package [52] developed for the software R and were used to identify bouts within a bird’s immersion state time series as: 1) a probable active foraging bout (a series of wet/dry event transitions lasting less than the *BEC*), 2) a sustained flight bout (any dry event lasting longer than the *BEC*) or 3) a floating bout (any wet event lasting longer than the *BEC*). We used individual *BEC*’s to delineate bout types within each bird’s immersion time series due to a high degree of individual-level variation (LAAL 33.5 ± 8.6 min and BFAL 45.6 ± 9.5 min, mean ± SD). Future studies applying this approach should assess *BEC* variation before proceeding to delineate bouts using either a single value across all birds [17] or assessing individuals independently (this study). From the bout-type classifications along the time series, daily activity parameters were calculated for each bird as noted above.

### Statistical analysis for day-to-day activity patterns

Data exploration indicated potentially non-linear relationships in daily activity parameters with time, thus we implemented generalised additive mixed models (GAMMs) to assess patterns in daily activity budgets over the course of non-breeding [37]. Due to a large number of zeros in the data, a two stage hurdle model was used to analyse sustained flight as either: 1) the time when birds were detected to be in sustained flight (proportion of sustained flight) or 2) whether birds were in sustained flight (flight: yes/no). As a smoothing function, this model included days since departure (DSD) from the colony. The time spent while floating on the water and actively foraging was used as the response variables for two additional models. Fixed categorical factors for all models included non-breeding phase and species. Individual bird was modelled as a random effect (intercept-only) as birds contributed repeatedly and unevenly with respect to data [53]. Adequacy of model fit was examined via autocorrelation lag plots, variograms, and the normalized residuals against independent variables including those not in the models (e.g. spatial location). Because our data consisted of a time series and were found to be autocorrelated, we included a temporal correlation structure (corExp, which also then accounted for associated spatial autocorrelation as positions close in time are also close in space, [54]). Including a correlation structure and random effect allowed us to model compound correlation between observations from the same bird and the temporal correlation between all observations from the same bird and DSD [54]. Backward model selection was performed until all terms were significant, and the correlation structure and random effect improved model fit for all three response variables. Models were again validated using the techniques described by [53].

## Results

### Overwinter movements and destinations

From colony departure to return, LAAL travelled on average 22,134 ± 3,825 km (mean ± SD, range 17,000-30,000 km). Total distance travelled ranged more widely for BFAL (17,997 ± 4,688 km, mean ± SD, range 11,000-28,000 km). Outbound and inbound transit phases were clearly identifiable for all birds, lasting 2 to 16 days for LAAL and 2 to 20 days for BFAL (Table 1). Periods of limited localized movements during the *c.* 125 days of overwinter were contained within one to three distinct areas for each bird with larger movements between areas lasting two to five days. For LAAL, birds were found mostly within the following three main regions (25% UD contour, Figure 1): (1) 75% of birds (15 of 20) ranged between the southern tip of the Kamchatka Peninsula, Russia, to the Commander Islands and the western side of the mid-to-northern Emperor Seamount, (2) 60% of birds used areas between 300–1000 km east of Honshu Island and Hokkaido Island, Japan and, (3) 30% ranged south of the southern-most islands of the Southern Aleutian Arc, Alaska. Three individuals spent 5–14 days in the pelagic mid-North Pacific to the northwest of the colony as their second or third overwinter destination, and one individual spent the first 73 days around the Aleutians before moving 1000 km W of the Oregon coast for 33 days. For LAAL that used only a single overwinter area (*n* = 4), two spent all of their time around the Kamchatka Peninsula and two east of the Japanese continental margin.

For BFAL, 53% (8 of 15) of birds spent at least some portion of the overwinter period centred around Unalaska Island of the Aleutians, ranging around 400 km north–south and 500 km east–west along the Alaskan Peninsula (25% UD contour, Figure 1); four birds remained in this region for the entire duration of the overwinter period. Another 53% of individuals spent time ranging comparatively widely across the mid-North Pacific, mostly north and northwest of the colony toward the Emperor Seamounts; three birds remained in this broad area making only localized movements throughout the overwinter period. Two BFAL used areas southeast of Honshu Island and Hokkaido Island, Japan, while one individual spent 30 days off the SW coast of Vancouver Island, BC, Canada, then 35 days in the Gulf of Alaska before finishing the overwinter phase in the mid-eastern North Pacific.

### Seasonal activity patterns

**Figure 2:**
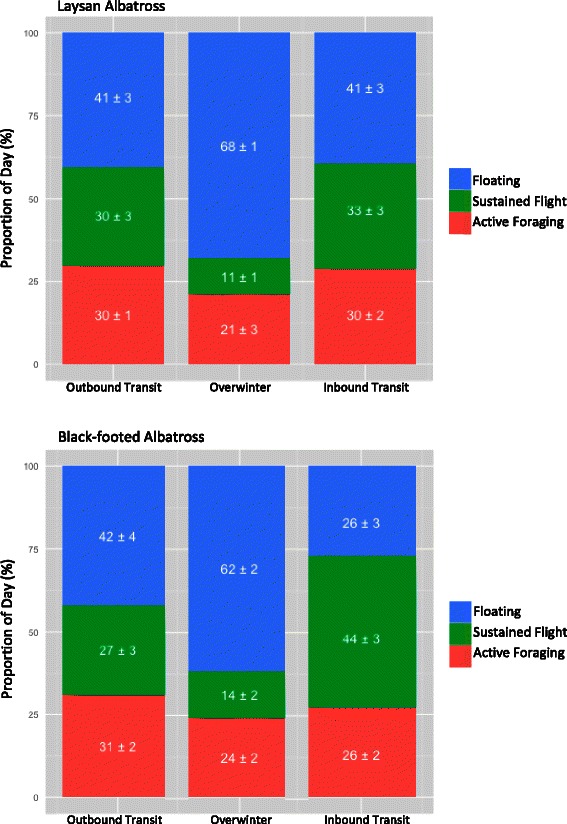
**Non-breeding activity budgets by phase for laysan and black-footed albatross from Midway.** Activity budgets derived from immersion-logger data for laysan albatross (*n* = 18, top panel) and black-footed albatross (*n* = 15, bottom panel) during the non-breeding period. The proportion of each day within each phase of non-breeding spent engaged in three different activity bout types are reported as mean ± SE.

For both species, sustained flight bouts comprised a high proportion of inbound and outbound transit days; 27 to 44% of each day was spent engaged in 1 to 4 flight bouts lasting roughly 2 hours each (Table 2; Figure 2). During overwinter, limited time was spent in sustained flight each day (Table 2, Figure 2). For the entire overwinter phase, LAAL spent on average 46.9 ± 16 days without engaging in any bouts of sustained flight accounting for 37 ± 10% of each individual’s overwinter phase, and BFAL 52.3 ± 14 days (43 ± 14% of overwinter). The vast majority of time during overwinter was detected as long and frequent floating bouts for both species (Table 2; Figure 2). Floating also comprised a high proportion of the day throughout both inbound and outbound transit phases but with less frequent short bouts (Table 2; Figure 2). For all phases and both species, on average 21 to 31% of each day was spent engaged in active foraging split between 2 to 5 individual bouts (Table 2; Figure 2).

**Table 2:** **Summary of daily activity among three phases of non-breeding for laysan (** ***n*** **= 18) and black-footed albatross (** ***n*** **= 15) from Midway Atoll (mean ± SD)**

	**Laysan albatross**			**Black-footed albatross**		
	**Out**	**OW**	**In**	**Out**	**OW**	**In**
Sustained flight bouts (/day)	2.8 ± 1.0	1.2 ± 0.3	3.4 ± 1.3	2.3 ± 1.0	1.2 ± 0.5	2.8 ± 1.1
Floating bouts (/day)	2.9 ± 1.4	3.4 ± 0.8	2.3 ± 1.0	2.0 ± 0.7	2.5 ± 0.5	1.5 ± 0.6
Active foraging bouts (/day)	4.3 ± 1.1	3.4 ± 0.7	4.3 ± 1.5	3.9 ± 1.1	2.8 ± 0.7	3.0 ± 1.2
Flight bout length (mins)	105 ± 32	67 ± 18	114 ± 27	114 ± 36	77 ± 20	120 ± 37
Float bout length (mins)	169 ± 65	264 ± 56	133 ± 55	270 ± 123	315 ± 63	175 ± 87
Forage bout length (mins)	77 ± 20	65 ± 16	75 ± 30	103 ± 18	84 ± 20	95 ± 35
Distance travelled (km/day)	285 ± 19	105 ± 4	433 ± 19	273 ± 22	76 ± 9	305 ± 48

Out = outbound transit, OW = overwinter, In = inbound transit.

### Daily activity patterns

**Figure 3:**
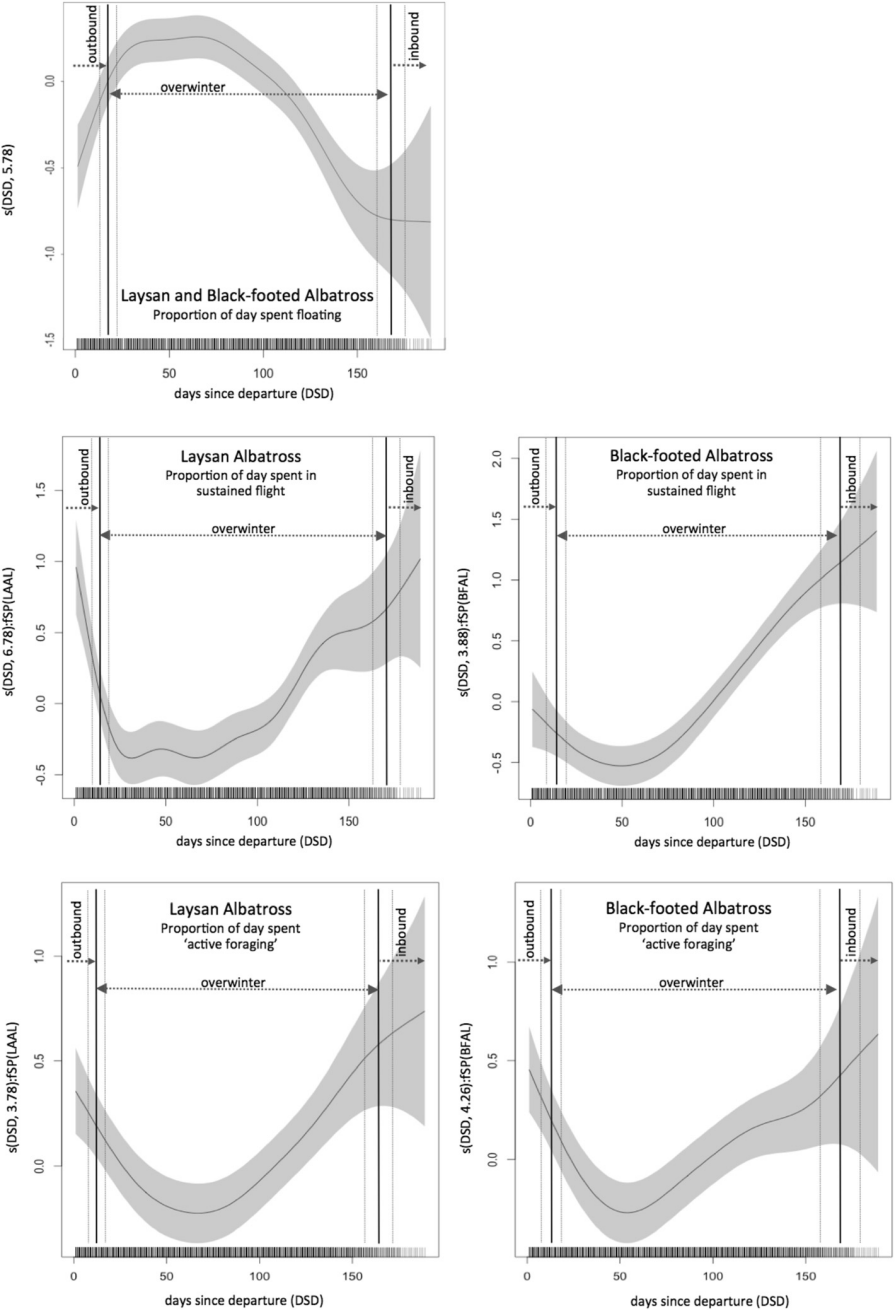
**Non-breeding season patterns in daily activity of laysan and black-footed albatross from Midway.** Partial residual plots of daily patterns in non-breeding activity for laysan and black-footed albatross. Estimated smoothing functions (solid lines) with 95% point-wise confidence intervals (delineated by the grey shaded area) estimated from the proportion of daily time spent floating on the water’s surface (top panel), engaged in sustained flight (middle panels) and actively foraging (bottom panels) smoothed by the days since colony departure (DSD). The relationship differed significantly between species for sustained flight and active foraging bouts although the general pattern over time is similar. Vertical lines depict the average duration of each non-breeding phase, with outbound transit followed by arrival day at the first overwinter area, and then inbound transit initiation (mean ± SD).

For both species, the proportion of each day spent in sustained flight followed a similar overall pattern with increasing DSD, but differed significantly in their smooth functions (Table 3; Figure 3). Both species showed an initial decrease in time spent in sustained flight over the first 30 days. LAAL exhibited a more rapid decline followed by an extended period of few daily flight bouts before increasing again. This differs slightly from the more gradual decline in daily flight time for BFAL, which reached a low around 50 days before gradually rising once more (Figure 3). Neither species displayed a noticeable shift in flight activity upon initial arrival at the first overwinter area, but instead steadily decreased time in sustained flight following arrival. The same pattern held true for the initiation of colony return for BFAL; these birds steadily increased the time spent in sustained flight bouts each day after the low-point in flight activity, gradually increasing flight time before and during their inbound transit journey. LAAL exhibited a slight rise in flight activity within ten days of the initiation of inbound transit, but overall show a less smooth but consistent pattern between species of increased flight activity following an approximately 40-day window of low flight activity from 30–70 DSD.

**Table 3:** **Results from the generalized additive and linear mixed-effects components of the GAMM output**

**Model #**	**Response**	**Model term**	***df***	***F***	***P-value***
1.	Sustained flight, >0	s(DSD):sp(LAAL)	6.78	12.82	< 0.0001
		s(DSD):sp(BFAL)	3.88	39.97	< 0.0001
		Non-breeding Phase	2	9.86	< 0.0001
		Species	2	15.98	< 0.0001
		Phase:Species	2	4.01	0.0181
2.	Sustained flight (0,1)	s(DSD)	4.50	23.73	< 0.0001
		Non-breeding Phase	2	10.07	< 0.0001
3.	Floating	s(DSD)	5.78	16.83	< 0.0001
		Non-breeding Phase	2	11.26	< 0.0001
4.	Foraging	s(DSD):sp(LAAL)	3.12	7.67	< 0.0001
		s(DSD):sp(BFAL)	3.58	6.19	< 0.001
		Non-breeding Phase	2	6.55	0.0014

Degrees of freedom for the smoothers are taken from the model hat matrix. Proportion of time spent daily in sustained flight was zero-inflated (>35% zeros) and thus was modelled in two parts as a hurdle model with both quasi-binomial and binary distributions (Models 1 and 2).

The temporal pattern in time spent engaged in floating bouts over the non-breeding period did not differ significantly between species (Table 3; Figure 3). An approximate 40-day window from 30–70 DSD also coincided with the highest proportion of time on the water’s surface. Again, the proportion of each day spent on the water continually increased before and after arrival at the first wintering area. After 70 days, all birds began to slowly decrease the proportion of each day floating until inbound transit began; at which point the amount of time floating each day reached a low but consistent level. The pattern in time spent active foraging for both species mirrored closely that seen for time spent in sustained flight as a similar overall pattern with increasing days since colony departure, but differing significantly in smooth functions between species (Table 3; Figure 3). Daily time spent engaged in active foraging activity gradually declined until a low around 60 DSD for LAAL and around 10 days earlier for BFAL, before rising once again. A period of low active foraging activity is again detectable roughly between 30–70 DSD for both species.

**Figure 4:**
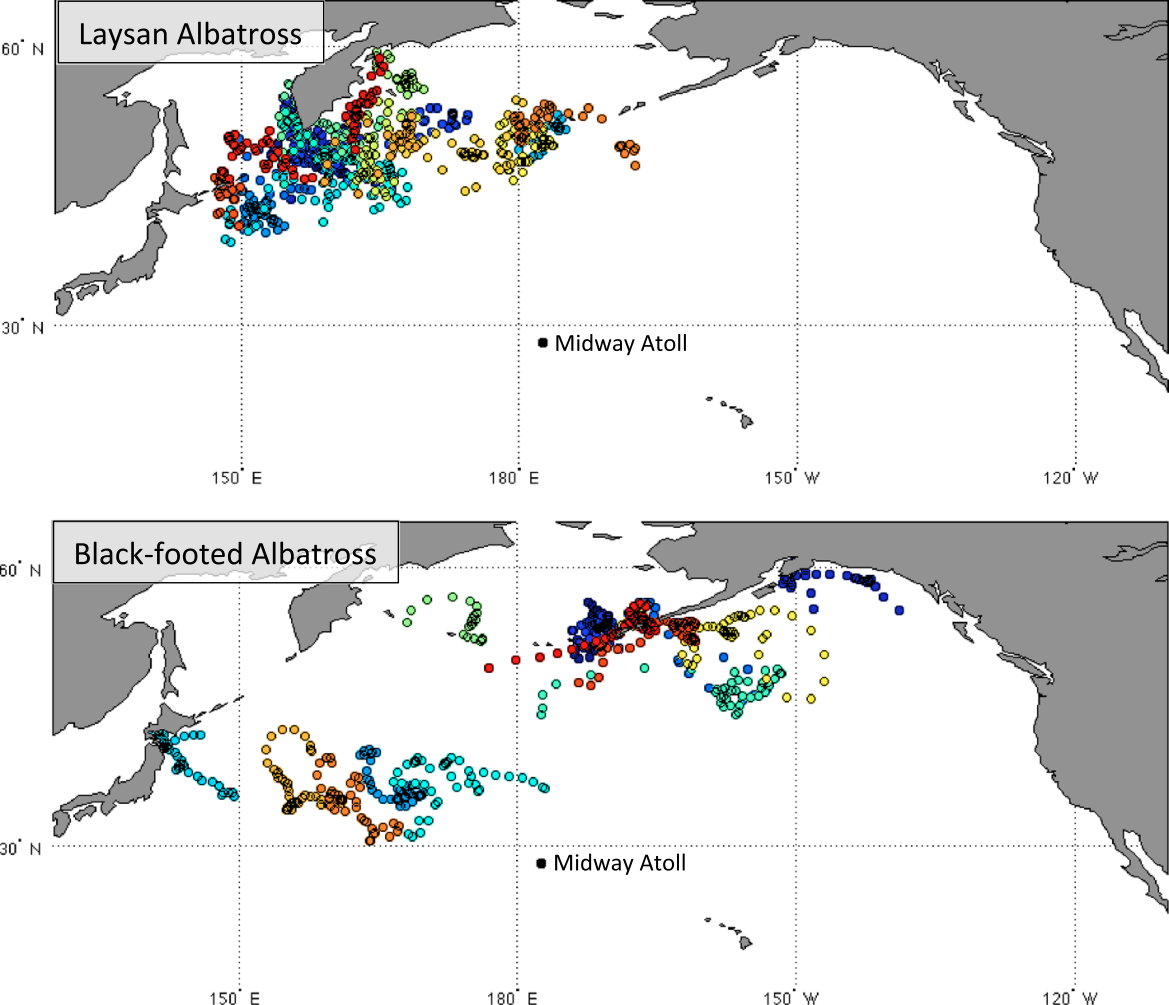
**At-sea distributions of laysan and black-footed albatross during the ‘quasi-flightless’ stage of overwinter.** Individual GLS-tracked laysan albatross (*n* = 20, top panel) and black-footed albatross (*n* = 15, bottom panel) during the ‘quasi-flightless’ stage of the overwinter phase (40-day window between 30–70 days since colony departure) in 2008, 2009, 2011 and 2012. Individual birds are indicated with unique colours.

The date of overwinter arrival and departure, and thus outbound and inbound transit phases, were determined based on spatial data, whereas the consistent pattern in activity between 30–70 DSD emerged from immersion-state activity budgets. We re-visited the spatial data within this window to examine whether the distribution of birds at-sea during this period differed from that of the *c.* 125-day overwinter phase as a whole (Figure 4). Indeed, the range of nearly all individuals during this time remained restricted within one of the previously identified overwintering areas; no birds made directed movements between major overwinter areas within this window. While all LAAL were confined to a small area of the northwest Pacific relative to the broader distribution of BFAL (Figure 4), the average daily distance travelled by individual LAAL was 77 ± 18 km/day, and by BFAL was 61 ± 26 km/day.

**Figure 5:**
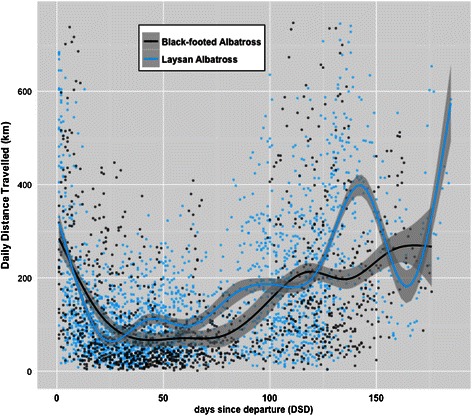
**Daily distance travelled (km) during non-breeding for laysan and black-footed albatross from Midway.** All raw data of daily distance travelled (km) from colony departure (DSD = 0) to return (varies by individual) from GLS-tracks of laysan albatross (*n* = 20) and black-footed albatross (*n* = 15) during the non-breeding season. A LOESS smoother was added to aid visual interpretation.

The number of days between 30–70 DSD with complete absence of sustained flight bouts detected was 21 ± 5 days for LAAL (ranging from 13–30 days) and 25 ± 6 days for BFAL (ranging from 14–33 days). All birds of both species spent at least one full day during this time entirely floating on the water. Further, LAAL on average spent 7 ± 5 days floating on the water’s surface for >90% of the day and 16 ± 7 days floating for >80% of the day. Similarly, BFAL on average spent 10 ± 7 days floating for >90% of the day and 18 ± 7 days floating for >80% of the day. In the time following this 40-day window until the birds initiated return inbound transit, LAAL travelled on average 50 km further each day, and BFAL 23 km each day (LAAL, 127 ± 27 km/day over 64 ± 19 days; BFAL, 84 ± 45 km/day over 66 ± 18 days) but this average value represents highly variable daily travel distances which generally increased following 70 DSD until colony return for both species (Figure 5).

## Discussion

Our study is the first we know of to examine seabird behaviour over the course of non-breeding at a detailed daily time-scale, allowing new insights into the modification of daily activity budgets as constraints on time and energy shift through this demanding life history phase. We also document associated movements and habitat use across the North Pacific Ocean basin, revealing distinct areas important throughout overwinter for both LAAL and BFAL. Over two-thirds of worldwide LAAL and one-third of BFAL return to the Midway Atoll colony to breed each year [22]. Our work, while restricted in sample size, adds to a limited body of research (i.e., [34]) explicitly examining at-sea habitat use and behaviour of these ‘Near Threatened’ residents [24] at any time in the breeding or life cycle.

### Overwinter destinations

During overwinter, LAAL and BFAL from Midway revealed discrete patterns in distributions throughout the North Pacific Ocean (Figure 1). Not surprisingly, these movements are associated with areas of known localized current convergence and upwelling that promote high primary and secondary productivity thus attracting fish, squid, and ultimately LAAL and BFAL [55]. Differences in habitat use among species were also expected and mostly follow that known from tracking studies of birds captured at-sea and from other smaller colonies throughout the annual cycle [25-34]. There were however some notable exceptions in the use of the Russian Kamchatka Peninsula region [29,31,32], California Current System [30,32] and more pelagic areas [29,31]. Together, the known distributions of non-breeding LAAL and BFAL indicate that these species range widely across the North Pacific during the four months when not tied to the colonies, crossing through multiple national and international jurisdictions and well into the high seas, with high individual- and colony-level variation in the use of broad overwinter areas. Future work should investigate variation within and between breeding colonies spanning the entire annual cycle of these species as necessary next-steps in the complete assessment of spatial ecology and population dynamics [3,22].

### Activity during transit phases of non-breeding

Outbound and inbound transit lasted around 9 to 12 days, although this ranged predictably between individuals depending on colony proximity to the first and last overwinter areas. Non-breeding LAAL and BFAL spent less time in flight than breeding birds on foraging trips from Tern Island during the brooding period [10]. Although [10] simply summed the number of 3-second intervals where immersion loggers registered as dry (thereby including time in sustained flight and flights within active foraging bouts), the average daily proportion of time off the water’s surface (90%) still far exceeds the combined time in flight and active foraging at any point in the non-breeding season (Figure 2). Brooding birds likely spent most of their time in flight searching out widely dispersed prey within close proximity to the colony. Migrating birds can rest more frequently and avoid areas of low productivity by adopting an opportunistic ‘fly-and-forage’ strategy similar to that reported for other migrating tubenoses (e.g. cory’s shearwater *Calonectris diomedea* [17]) and migratory birds of prey (e.g. osprey *Pandion haliaetus* [56]).

### Daily activity patterns during overwinter

It has been suggested that floating may comprise the vast majority of time during overwinter due to relatively low energetic requirements that are readily met while free from central-place constraints and chick-provisioning demands [14]. For example, comparable maximum flight bout durations during breeding and non-breeding in four species of southern hemisphere albatrosses could indicate that movement is not restricted but that birds are exercising the freedom afforded by low energetic demands to rest after directed movements between profitable foraging areas [14]. Our results suggest that while infrequent but long flight bouts during non-breeding may be similar in duration to those taken during breeding, the proportion of each day spent engaged in different activity types and the daily distances travelled are still likely to differ, especially if non-linear day-to-day temporal shifts in activity are considered. Differences in average activity budgets between overwinter and transit phase days did not reflect immediate modifications to daily activity budgets upon arrival to overwintering areas, but instead masked a gradual shift in activity toward a ‘quasi-flightless’ stage (where birds appear to be flight-limited though not completely) followed by an increasing trend in flight and active foraging until colony return (Figure 3).

The ‘quasi-flightless’ stage is matched by highly restricted ranges and daily movements of individual birds (Figures 4 and 5) and coincides with a known period of intensive flight feather moult and loss of body fat stores [57]. The sandy breeding habitat of LAAL and BFAL causes severe abrasion to the outermost primary flight feathers, leading to P8-P10 replacement annually overwinter, and an overall complex moult strategy [58]. The most intensive moult (all four series) causes 25% of LAAL and BFAL to skip breeding in the following season; time and energy are too limiting to accomplish both [59]. All of the birds in this study returned to Midway and were captured on the nest, so we assume none of these birds underwent a complete intensive moult but that all replaced at least their first three primaries along with initiating one or two other moult series during the ‘quasi-flightless’ stage of overwinter.

Approximately 40–60 days are required to complete moult during which at least one to three feathers within each series of each wing are missing or growing at any time [57,58]. Albatross have highly specialized anatomy for exceptionally efficient gliding flight, where rigid feather “sails” on long, slender, pointed wings are supported by specially adapted wing muscles and joints [23,60]. Worn, missing and growing feathers can compromise the wing’s airfoil through fluttering, creating asymmetries in wing shape and aspect ratio, and increasing wing loading from decreased wing surface area [61-64]. Lower body mass during moult may aid lower wing loading [57], but this likely does not compensate for increased flight costs given the high sensitivity of albatrosses to even small reductions in flight dynamics [64]. Added flight and feather synthesis costs likely constrain birds from relying heavily on ‘active foraging’ or ‘fly-and-forage’ strategies and from engaging in long bouts of soaring flight. Occasional larger movements may occur when the benefit of travelling from a crowded or poor foraging area outweighs the cost of flight, when small moult extents are accomplished more quickly for some individuals, or when strong currents simply carry floating birds away from a particular region (Figures 4 and 5). Effectively, both LAAL and BFAL likely experience *c.* 40 days of facultative quasi-flightlessness where foraging strategies shift to predominantly ‘sit-and-wait’ tactics.

Similar U-shaped temporal patterns in overwinter foraging activity have been documented in other tubenoses (e.g. manx shearwater *Puffinus puffinus* [19]). Birds may be intensely foraging after initial arrival to the overwintering grounds, possibly to replace body condition lost during breeding and to build up energy and nutrients needed for upcoming feather replacement [15]. Following the ‘quasi-flightless’ stage, LAAL and BFAL may begin a ‘post-moult rush’ to gain mass in preparation for breeding. Moult status and fat scores of drowned birds salvaged from drift-net fisheries showed a marked increase in body condition from relatively low fat stores during active moult to significantly higher following moult termination (10-20% gain in body mass [57]). Further, other albatrosses initiate egg formation *c.* 30 days before colony arrival [65], and both sexes of LAAL and BFAL are known to arrive to the Midway colony with extensive fat deposits and females with eggs in the oviducts [66]. The period of rapid fat accumulation and probable egg formation coincides with the gradual increase in sustained flight and active foraging along with less time floating on the water as colony return approaches (Figure 3). This ‘pre-migratory hyperphagia’ suggested for some other tubenoses (e.g. cory’s shearwater [17]) appears to progress steadily following the ‘quasi-flightless’ stage (Figures 3 and 5) and is likely a crucial ‘post-moult rush’ for breeding preparation as birds become increasingly mobile and actively seek out fruitful foraging areas before departing on their inbound transit journey.

## Conclusions

Importantly, the shift in activity budgets and habitat use within the overwinter period would have gone undetected if patterns were not assessed at a daily time-step relative to individual-level DSD from the colony. Other tubenose species, even those that tend to replace primary feathers biennially during non-breeding, may also exhibit identifiable stages during non-breeding if patterns in daily activity are examined at a daily temporal resolution. Because colony departure dates varied (across 54 days for LAAL and 39 days for BFAL), the calendar days when individuals at sea are undergoing these drastic adjustments to activity budgets span half the year, from June to November. The oceanic areas important during overwinter spread across nearly the entire North Pacific Ocean basin for birds from the large Midway colony, and likely further still into the California Current for birds from other colonies [30,32].

For LAAL from Midway, the Northwest corner of the Pacific Ocean is clearly a critical area for the potentially vulnerable ‘quasi-flightless’ stage (Figure 4) and for nearly all birds at some point during non-breeding (75% of LAAL in this study used this region for at least one of three overwinter areas). These waters must offer immense productivity to support birds mostly feeding opportunistically while floating on the water’s surface. Many other non-breeding tube-nosed seabirds also target this area including the ‘Vulnerable’ short-tailed albatross *Phoebastria albatrus* [67] and several trans-equatorial migratory shearwater species [7,68,69]. The productive Russian Far East is also the focus of an industrial demersal long-line fishery estimated to kill an average 6,500 seabirds/year, making the Russian Exclusive Economic Zone a prime candidate for marine protective measures [70]. In contrast, individual BFAL from Midway are spread widely across the North Pacific during this time but individuals tend to remain in relatively localized areas, likely with sufficient resources for meeting the nutritional demands of feather replacement and days spent mostly on the water (Figure 4). The wide distribution of BFAL may buffer against potential threats during this vulnerable time, but would pose a challenge to targeted protected areas.

It is well accepted that events occurring outside of breeding critically influence the demography of migratory populations [3]. The restricted distributions and modifications to activity during the non-breeding period for LAAL and BFAL are likely at least in part due to energetic constraints imposed by the necessity of plumage replacement. This may be even more pronounced in birds that skip breeding to undergo complete moult extents [59]. For at least a 40-day window of each year, these birds are relegated mostly to the ocean’s surface. This is probably to recover from and prepare for the taxing demands of an extreme life history strategy leaving little time to refresh flight feathers critical to their long-distance oceanic travels. Clearly, far-ranging migrants must carefully manage trade-offs in the allocation of limited time and energy toward shifting energetic demands as primacies shift throughout distinct life history phases and also at a finer day-to-day scale within these periods.

## Abbreviations

LAAL: Laysan albatross *Phoebastria immutabilis*
BFAL: Black-footed albatross *Phoebastria nigripes*
DSD: Days since (colony) departure (for non-breeding season initiation)
